# Single-molecule RNA sequencing for simultaneous detection of m6A and 5mC

**DOI:** 10.1038/s41598-021-98805-z

**Published:** 2021-09-29

**Authors:** Takahito Ohshiro, Masamitsu Konno, Ayumu Asai, Yuki Komoto, Akira Yamagata, Yuichiro Doki, Hidetoshi Eguchi, Ken Ofusa, Masateru Taniguchi, Hideshi Ishii

**Affiliations:** 1 The Institute of Science and Industrial Research, Osaka University, 8-1 Mihogaoka, Ibaraki, Osaka 567-0047 Japan; 2 Center of Medical Innovation and Translational Research, Graduate School of Medicine, Osaka University, 2-2 Yamadaoka, Suita, Osaka 565-0871 Japan; 3 Artificial Intelligence Research Center, The Institute of Scientific and Industrial Research, Osaka University, 8-1 Mihogaoka, Ibaraki, Osaka 567-0047 Japan; 4 Prophoenix Division, Food and Life-Science Laboratory, Idea Consultants, Inc., 1-24-22 Nanko-kita, Suminoe-ku, Osaka-city, Osaka 559-8519 Japan; 5 Gastroenterological Surgery, Department of Surgery, Graduate School of Medicine, Osaka University, 2-2 Yamadaoka, Suita, Osaka 565-0871 Japan

**Keywords:** Nanobiotechnology, Sequencing, Analytical chemistry, RNA, Nanoscience and technology, Analytical biochemistry

## Abstract

Epitranscriptomics is the study of RNA base modifications involving functionally relevant changes to the transcriptome. In recent years, epitranscriptomics has been an active area of research. However, a major issue has been the development of sequencing methods to map transcriptome-wide RNA base modifications. We have proposed a single-molecule quantum sequencer for mapping RNA base modifications in microRNAs (miRNAs), such as N6-methyladenosine (m6A) or 5-methylcytidine (5mC), which are related to cancer cell propagation and suppression. Here, we investigated 5mC and m6A in hsa-miR-200c-5p extracted from colorectal cancer cells and determined their methylation sites and rates; the data were comparable to those determined by mass spectrometry. Furthermore, we evaluated the methylation ratio of cytidine and adenosine at each site in the sequences and its relationship. These results suggest that the methylation ratio of cytidine and adenosine is facilitated by the presence of vicinal methylation. Our work provides a robust new tool for sequencing various types of RNA base modifications in their RNA context.

## Introduction

The epitranscriptome refers to RNA base modifications that occur after transcription^1^. There are about 130 types of known RNA base modifications in eukaryotic cells^2^, most of which occur in transfer and ribosomal RNA^3,4^. These RNA modifications play various important roles in the structures of transfer and ribosomal RNA. Recently, RNA base modifications have been identified in messenger and microRNA (mRNA and miRNA, respectively); these modifications include N6-methyl-adenosine (m6A), 5-methyl-cytosine (5mC), inocine, pseudouridine, 2′-O-methyl-base, and N6,2′-O-methyl-adenosine^5–11^. Some of these RNA base modifications are known to enhance the stability and translation efficiency of mRNA^7^.

In miRNA, RNA base modifications play roles in miRNA processing and target suppression efficiency^8^, resulting in functional changes. Therefore, information about the types and quantities of RNA base modifications is important. Some RNA base modifications have been detected using RNA immunoprecipitation coupled with sequencing^12^. This method uses antibodies specific for particular base modifications. Therefore, only one type of RNA base modification can be detected in a single sequence. Actually, there are currently very few technologies for multiple types of base modifications present on a single RNA strand by MASS-spectroscopy and chemical sequencing method including bisulfite sequencing^13^. Therefore, the development of a comprehensive analytical technology to simultaneously detect various base modifications in a single RNA strand will be an important tool for enhancing our understanding of the epitranscriptome^14,15^.

Single-molecule sequencing is emerging as a method for understanding the RNA epitranscriptome because it can detect the physical properties of nucleobases in a sample nucleotide; thus, it has the potential to detect the location of any base modifications without PCR amplification, including methylations such as 5mC^16–20^. We have previously reported a single-molecule electrical resequencing method using nano-gap devices^21–26^. This methodology is based on sequentially reading the tunneling currents across individual single nucleotides in the sequence, resulting in high-speed electrical discrimination between individual nucleotides without chemical probes and PCR amplifications. Each base conductance value is determined by the individual molecular energy level of each of DNA/RNA nucleotide. Therefore, this method could identify any kind of known and/or unknown modified nucleotide, allowing comprehensive epigenetic analysis of the genome and transcriptome.

**Figure 1:**
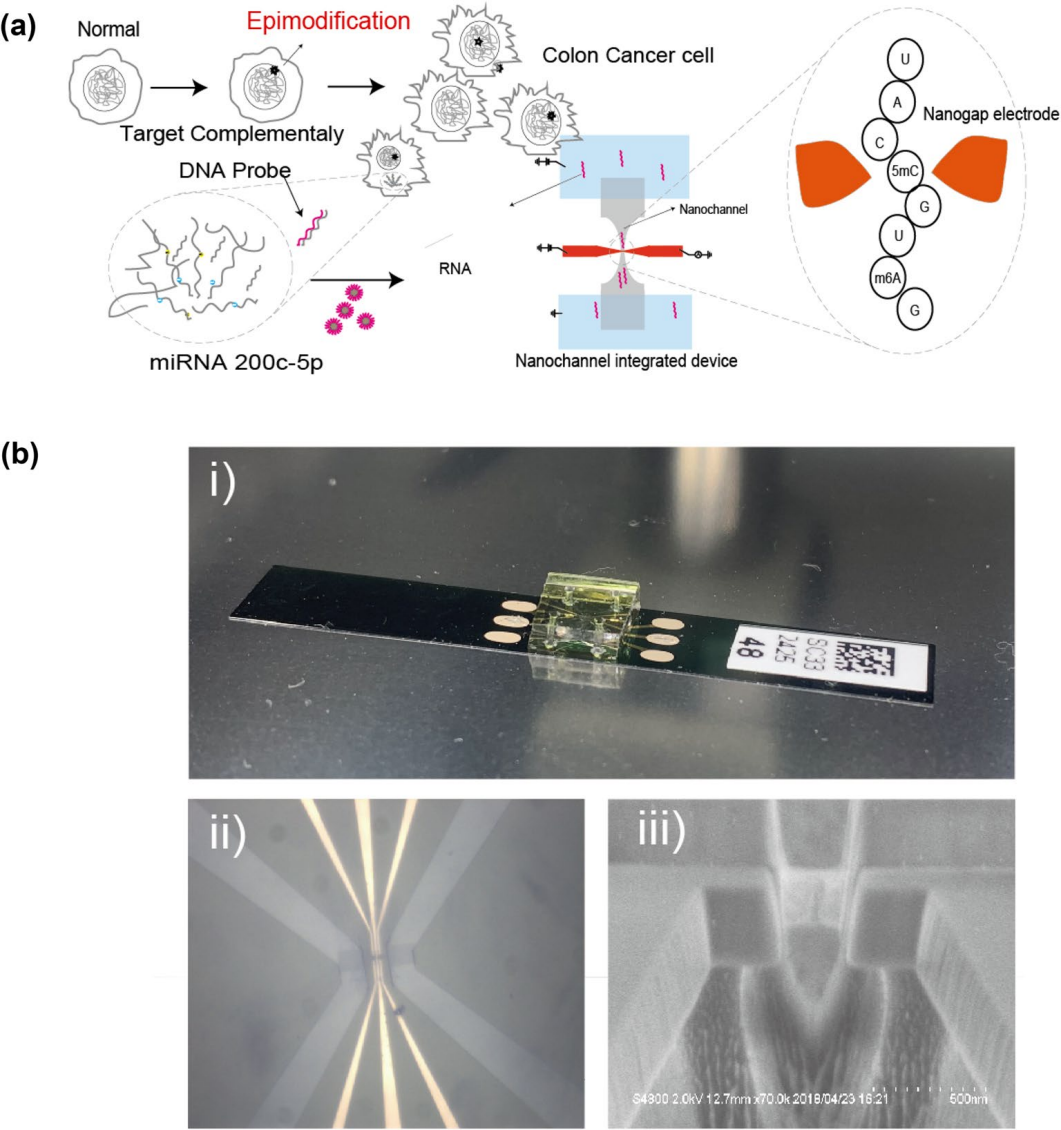
Single-molecule miRNA epitranscriptome analysis. (**a**) Flow chart of epi-miRNAseq by single-molecule electrical quantum sequencing. (**b**) (i) Photo of the nanochannel integrated nano-gap device. The substrate is 50 mm long and 8 mm wide. The device comprises a silicon substrate fused with a PDMS cover, which has a microchannel and solution chambers (bottom right). (ii) An optical image (100 μm × 100 μm) of the PDMS cover fused to the nanochannel integrated nanogap device is shown. The microchannel of the PDMS cover is connected to the nanochamber regions of the silicon substrate, which have squired-pillar regions in the chamber to prevent the ruff-craps of PDMS. (iii) SEM image of a nanochannel integrated nano-gap device, which has a nano-gap electrode and a nanochannel near the nano gap.

In this study, we investigated multiple types of RNA modifications in miRNA by using single-molecule electrical resequencing of miRNAs extracted from colorectal cancer cells (Fig. 1a). We focused on detections of the modified nucleotide sites 5mC and m6A, which are typical epigenetic modifications in miRNA and naturally generated by various methyltransferases. We evaluated the ratios of methylated cytidine to non-methylated cytidine and methylated adenosine to non-methylated adenosine in miRNA and its relationship. These results suggest that the methylation ratios of cytidine and adenosine are facilitated by the presence of vicinal methylation. Our technique will allow for greater understanding of the epitranscriptome by enabling the sequencing of various RNA base modifications in their RNA context.

## Results and discussion

### Determination of m6A and 5mC conductivity using single-molecule electrical detection

We investigated methylation site detection of synthesized RNA nucleotides using a single-molecule electrical resequencing method. The detection scheme was as follows (Fig. 1a) (Supporting information (SI): S1–S6, Figs. S1–S3). In the first step, the conductivity of each nucleotide in the synthesized RNA molecules was measured by sequentially reading across individual single nucleotides with nano-fluid integrated nano-gap devices (Fig. 1b), in which the nano-fluid strongly confined nucleotide translocation, resulting in guidance of the nucleotide molecules straight into the fluid region under direct current (DC) voltage across the gap electrode. The obtained conductance–time profiles represented the conductance sequence of each nucleotide in the synthesized oligonucleotide translocated through the gap electrodes. In the second step, the Phred base-calling method^27,28^ was used for each of the conductance–time profiles based on the conductivity of mono-nucleotides (SI: S5–S7) and determined the sequences of the oligonucleotides being translocated through the gap electrodes (Fig. S5). In this base-calling method, the conductance profiles of any detectable types of nucleotides, including methylated ones, are required. Therefore, in this study, we re-measured the characteristic conductance profiles of 5mC and m6A (SI: S3, Figs. S1–S3) and determined the conductance values of 5mC and m6A for 105 picosiemens (10^−12^ S = pS) and 92 pS, respectively (SI: S2, and Fig. S2). Together with the previous conductance results of RNA mono-nucleotide^21^, we found that the order of the conductance values is as follows: 5mC (105 pS) > rGMP (87 pS) > m6A (92 pS) > rAMP (67 pS) > rCMP (60 pS) > rUMP (36 pS) (Table 1) (Fig. S4). These values were used for base calling in the second step. In the third step, the sequences determined were mapped by assembly against the original sample sequences (SI: S6, and Figs. S5, S6). Based on the mapped sequences, the conductance profiles were obtained, and the methylation ratios in the sample nucleotides were evaluated, especially each of the cytidine and adenosine sites in the sample nucleotides.

**Table 1:** Single-molecule conductance (*G*) and relative single-molecule conductance of ribonucleosides and epinucleosides (5mC and m6A).

Ribonucleoside/epinucleoside	Ribonucleoside/epinucleoside name	Conductance (pS)	Relative *G*
G	Guanosine	123	1
A	Adenosine	92	0.75
C	Cytidine	64	0.58
U	Uridine	50	0.41
5mC	5-Methyl-cytidine	149	1.21
m6A	6-Methyl-adenosine	111	0.90

The ratio of the number of methylated A bases to the total number of A bases in the detected fragmented miR-200c-5p signals is defined as the methylation ratio (%) for m6A/A in the second column of this table. Similarly, the ratio of the number of methylated cytidine bases to the total number of cytidine bases is defined as the methylation ratio (%) for 5mC/C. Each of the base numbers is counted from the base composition of the fragmented miR-200c-5p.

### Determination of m6A and 5mC modification ratios in miRNAs of colon cancer cells

**Figure 2:**
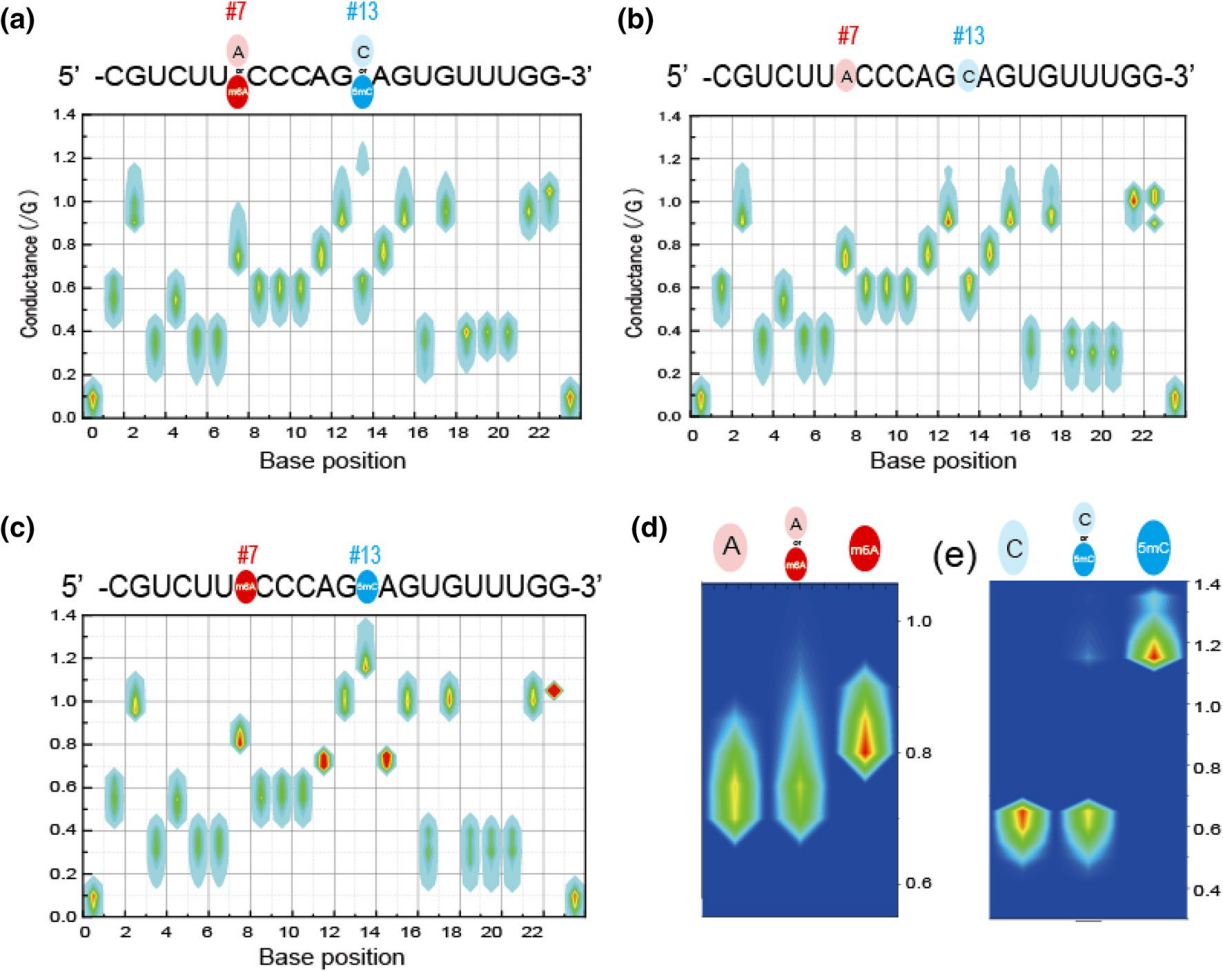
Determination of miR-200c-5p base sequence. (**a**) Heat maps of RNA conductance plots for miR-200c-5p extracted from colorectal cancer cells (DLD-1). (**b**) Heat maps of synthesized miR-200c-5p, in which adenosine and cytosine are non-methylated. (**c**) Heat maps of RNA conductance plots of synthesized miR-200c-5p in which adenosines #7 and #13 are methylated. The x and y axes are the base position and conductance normalized to the conductance of guanine, respectively. (**d**) Enlarged conductance plots of the #7 position adenosine for non-methylated miR-200c-5p (left), captured miR-200c-5p (middle), and methylated miR-200c-5p (right). (**e**) Enlarged conductance plots of the #13 cytidine for non-methylated miR-200c-5p (left), captured miR-200c-5p (middle), and methylated miR-200c-5p (right).

We previously reported that some RNA base modifications are enhanced in cancer cells compared with normal cells; in particular, the detection of m6A in miRNA would be helpful in pancreatic cancer diagnosis^6^. In this study, we applied this method for estimation of the methylation rate in sample miRNAs extracted from cells of a typical colorectal cancer cell line (DLD-1). Of the DLD-1 miRNAs, miR-200c-5p (5′-CGUCUUACCCAGCAGUGUUUGG-3′) is strongly associated with cancer progression and metastasis^29^. To measure RNA base modification levels in miR-200c-5p (SI: S9, and Fig. S7a), mature miR-200c-5p was extracted from total RNA and captured by a complementary DNA (cDNA) probe attached to magnetic beads before measurement of the conductance profiles. Figure 2a shows the conductance plots of the captured miR-200c-5p sample, which is constructed from 2000 conductance signals (SI: S3, and Fig. S2). The average conductance levels of the captured sample miRNA were in agreement with those of the non-methylated synthesized miR-200c-5p oligonucleotide (Fig. 2b,c). This suggests that most of the captured RNA was comparable to miR-200c-5p.

Importantly, in the miR-200c-5p conductance profiles, larger conductive signals around 1.2 relative G (normalized to the electrical conductance of guanine) coexisted with the conductance of cytidine around 0.6 relative G at the cytidine sites (Fig. 2e). Since the conductance levels of the larger signals were comparable to that of 5mC, the larger signals were likely due to 5mC signals (Table 1). The ratio of the 5mC signal number to the C signal number was found to be 4.6% (4809/103,924) (SI: S9, Fig. S7b). This suggests that 4.6% of cytidine in the miRNA was methylated, which is comparable to the 3.0% methylation rate found previously in small RNAs in HCT116 cells by liquid chromatography (LC)–tandem mass spectrometry (MS/MS).

Similarly, we found larger conductive signals around 0.9 relative G at the adenosine sites (Fig. 2d), which were comparable to that of m6A (Table 1). The ratio of m6A signal number to A signal number was 2.9% (1921/67,154), suggesting that 2.9% of adenosine in the sample miRNA was methylated. The methylation ratio determined by our method is comparable to the 1.2% ratio found previously in small RNAs in HCT116 cells by LC–MS/MS^1^.

### Dependence of 5mC modification rate on cytidine base position and consensus sequence

**Figure 3:**
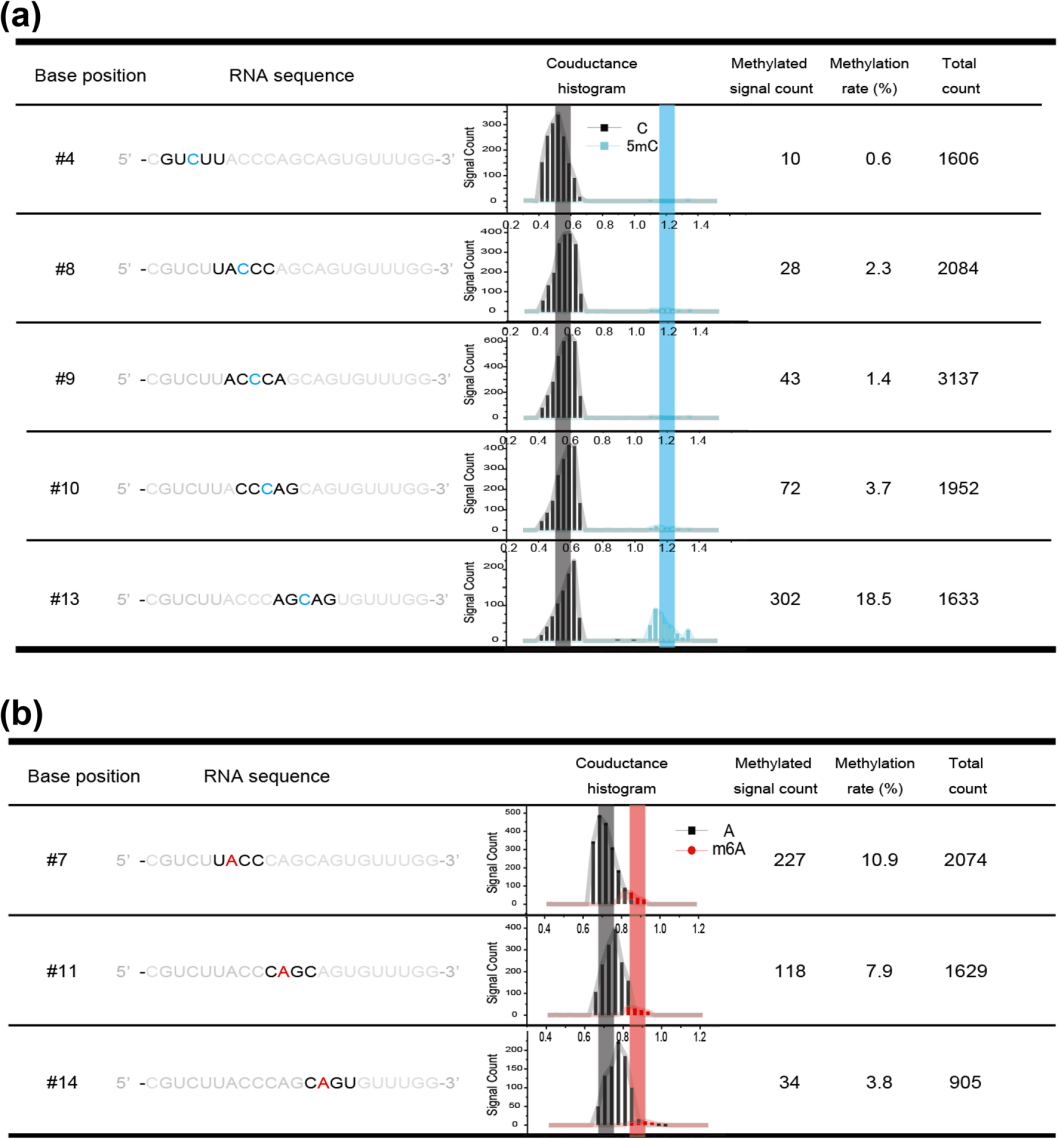
m6A and 5mC counts for single-molecule methylated miR-200c-5p. (**a**) 5mC modification rates in miR-200c-5p. In the second column, the sequences neighboring the methylated cytosines are shown. In the third column, the conductance histograms relative to those of guanine are shown. The black and blue lines represent the typical relative conductance values for C and 5mC, respectively (Table 1). (**b**) m6A modification rates in miR-200c-5p. In the second column, the sequences neighboring the methylated adenosines are shown. In the third column, the conductance histograms relative to those of adenosine are shown. The black and red lines represent the typical relative conductance values for A and m6A, respectively (Table 1).

We evaluated differences in the methylation ratios (5mC/C) of the cytidine sites (#4, #8, #9, #10, and #13) in the sample miR-200c-5p nucleotide by signal assembly of the right length of read sequence before and after the cytidine base number to be evaluated. The 5mC methylation ratio was the ratio of the number of methylated nucleotides to the number of non-methylated nucleotides for a given site. In the captured miR-200c-5p, 5mC/C was 0.6% for #4 (10/1606), 1.3% for #8 (28/2084), 1.4% for #9 (43/3137), 3.7% for #10 (72/1952), and 18.5% for #13 (302/1633) (Fig. 3a). This suggests that cytidine #13 of miR-200c-5p is highly methylated. The methylated fragment containing the #13 cytidine in miR-200c-5p was also detected by MALDI–TOF MS/MS (SI: S10, Fig. S8). Therefore, this method enables us to evaluate the methylation status of each of the cytidine sites in a miRNA.

The 5mC methylation of RNA is carried out by methyltransferases, such as NSUN2. The consensus sequences targeted by NSUN2 are CHG, CHH, and CG (H = A, C, or U)^30^. Of the cytidine methylation sites, #4 (CUU), #8 (CCC), and #9 (CCA) match the CHH consensus sequence, and #10 (CAG) and #13 (CAG) match the CHG consensus sequence. The methylation ratios for the CHH and CHG sites were 1.2% (81/6827) and 10.4% (374/3585), respectively. These results suggest that the methylation in the miR-200c-5p sample extracted from colorectal cancer cells was induced by the NSUN2 methyltransferase.

### Dependency of m6A modification rate on adenosine base position and consensus sequence

We also evaluated the differences in methylation ratios (m6A/A) between the adenosine sites (#7, #11, and #14). In the captured miR-200c-5p, the methylation ratios (m6A/A) were found to be 10.9% (227/2074) for #7, 7.2% (118/1629) for #11, and 3.8% (34/905) for #14 (Fig. 3b). This suggests that #7 is the most highly methylated adenosine site. The fragmented methylation of #7 adenosine in miR-200c-5p was also detected by MALDI–TOF MS/MS (SI, S5, Fig. S2). Therefore, this method enables us to evaluate the methylation status of each adenosine site in a miRNA.

It has previously been reported that the m6A methylation of RNA is caused by methyltransferases such as METTL3 complex^31^, and the consensus sequence that it recognizes is RACH (R = A or G; H = A, U, or C). A three-base match (matching rate: 75%) to the RACH consensus sequence was found in the sequence neighboring the methylation site of #7 (UACC), and a two-base match (matching rate: 50%) was found in those of #11 (CAGC) and #14 (CAGU). The similarities to the RACH consensus sequence of the sequences neighboring the methylated adenosines suggest that miR-200c-5p methylation was induced by a METTL3 methyltransferase complex.

### Correlation of m6A and 5mC

**Figure 4:**
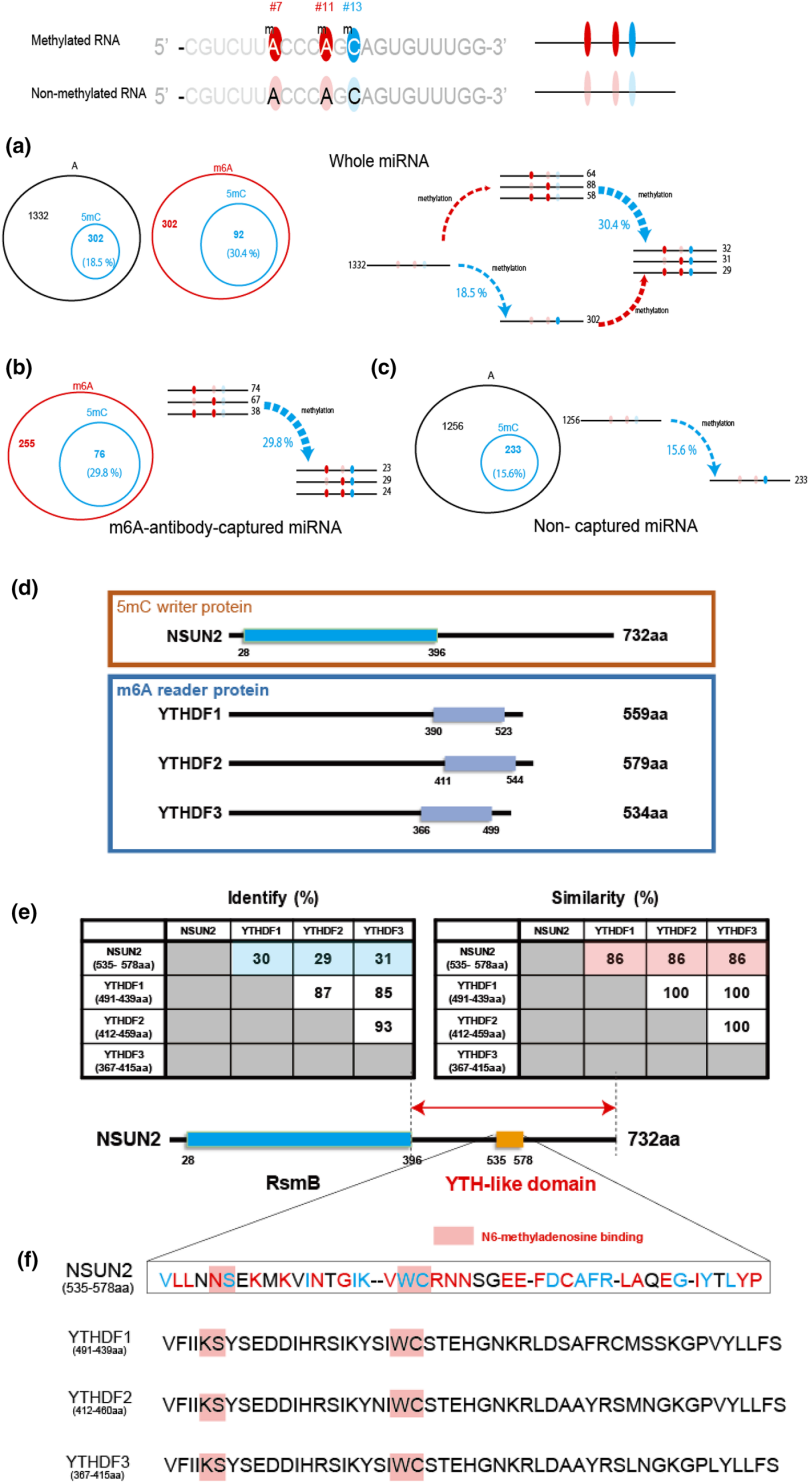

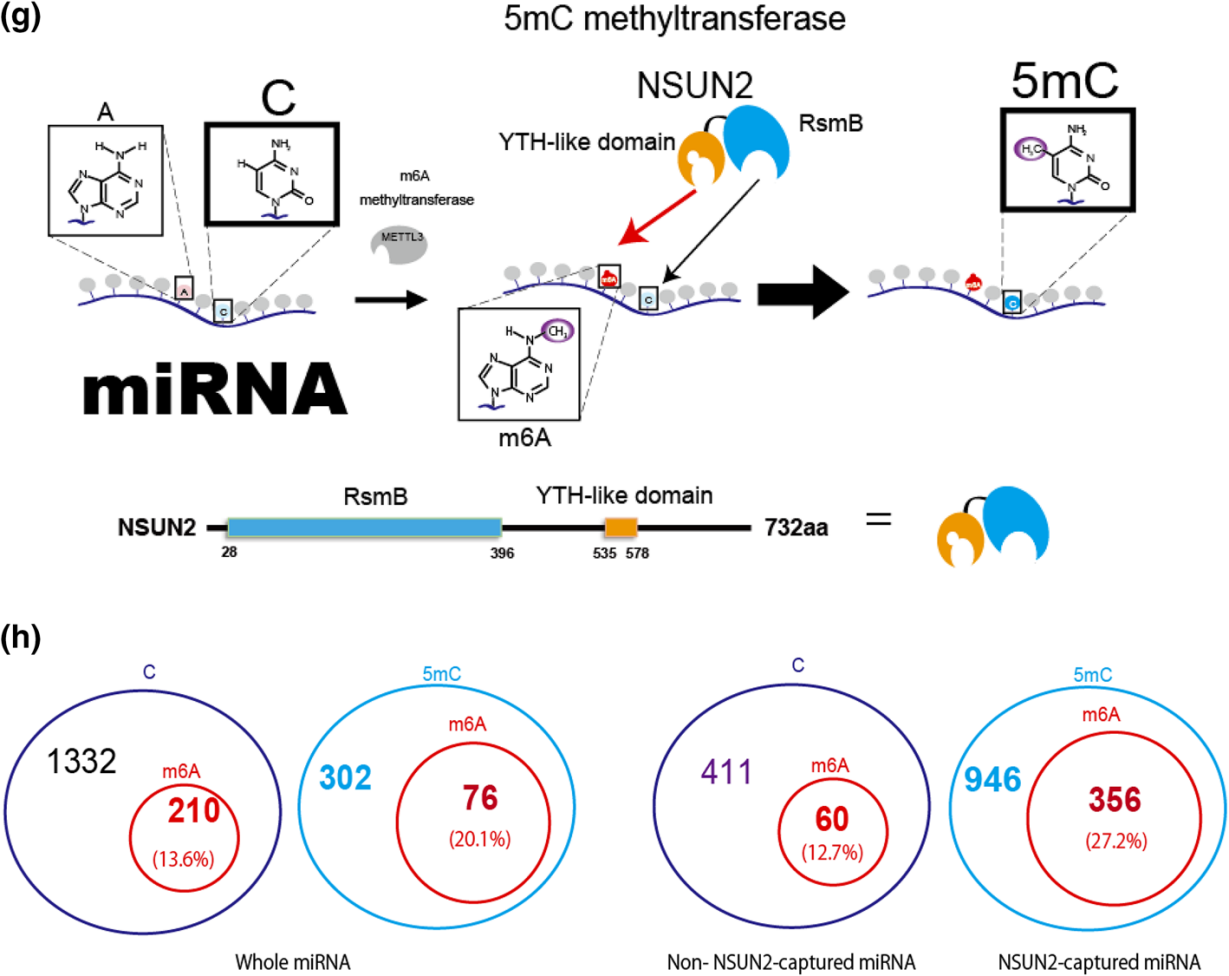
Proposed mechanism for formation of the miR-200c-5p epitranscriptome. (**a**) Mechanism of formation of the epitranscriptome in the whole miRNA. Epi-distribution for all 1729 signal numbers in the whole miRNA. The signal numbers for all sequence combinations containing non-methylated/methylated #7 (A), #11 (A), and #13 (C) are shown. (**b**) Epi-distribution for all 255 signal numbers in the m6A-antibody-captured miRNA, (**c**) Epi-distribution all 1,489 signal numbers of the miRNA in the supernatant after m6A-antibody capture (m6A non-captured miRNA). (**d**) Structures of NSUN2 and YTHDF proteins. The RsmB domain in NSUN2 is denoted by a green rectangle. The YTH domains are indicated by light blue rectangles. (**e**) Amino acid homology between NSUN2 and YTHDF YTH domains. (**f**) Amino acid comparison between the putative YTH domain of NSUN2 and the YTH domains of the YTHDF proteins. The amino acids shown in red match the YTH domain exactly, and the amino acids shown in blue have similar properties to the YTH domain. The amino acids in red squares recognize m6A. (**g**) Model of how miRNA 5mC modification is induced by m6A modification. (**h**) Epi-distribution for all 1729 signal numbers in the whole miRNA. (left two panels). The A-methylation rate (m6A/A) in 5mC-containing miRNA which binds NSUN2 was 20.1% (76/378) (first left panel). On the other hand, A-methylation rate (m6A/A) in miRNA which neither contain 5mC nor bind NSUN2 was 13.6% (210/1542) (second from the left panel). Epi-distribution for all 946 signal numbers in the NSUN2-captured miRNA, and for all 471 signal numbers in non-NSUN2 captured miRNA (right two panels). The A-methylation rate (m6A/A) in 5mC-containing miRNA which binds NSUN2 was 27.4% (356/1302) (first right panel). On the other hand, A-methylation rate (m6A/A) in miRNA which neither contain 5mC nor bind NSUN2 was 12.7% (60/471) (second from the right panel).

Finally, we evaluated the signals of A and C methylations coexisting on a single molecule. In this study, we focused on correlating the 5mC methylation of site #13 with m6A methylation (sites #7 and #11) because the 5mC methylation was highest at site #13 compared with the other 5mC modification positions (#4, #8, #9, and #10) in the miR-200c-5p sample, as shown in Fig. 3a and b, respectively. Figure 4a shows each methylation signal number and ratio for sites #7–13 of miR-200c-5p. Of the total 1936 signal numbers, the ratio of non-methylated signals to total numbers was 68.8% (1332/1936), and the m6A (#4 and #11) modified signal ratio was 10.8% (210/1936). The 5mC (#13) modified signal ratio was 15.6% (302/1936).

Importantly, both m6A and 5mC modified signal to total ratios were 4.8% (92/1936). This was the first time that simultaneous detection of m6A and 5mC in the same miRNA molecule had been achieved. These results suggest there is crosstalk between m6A and 5mC methylation. For instance, the ratio of 5mC methylation among m6A methylated signals was 30.4% (92/302), which was much larger than 18.5% of the ratio of 5mC methylated signals to the non-methylated signals (302/1634), and 20.3% of the 5mC modification to the total signal number (394/1936) (Fig. 4a). To confirm the methylation rates, we investigated the methylation rate of 5mC in RNA immunoprecipitated using an anti-m6A-antibody (targets m6A-containing total RNA) and non-captured RNA samples (RNA without m6A modifications). The C-methylation rate (5mC/C) in m6A-containing miRNA was 29.8% (76/255) (Fig. 4b) and 15.6% (233/1489) in the miRNA sample not containing m6A modifications (Fig. 4c); these values are comparable to 30.4% and 18.5%, respectively.

Together, these results suggest that the m6A modification of #7 and/or #11 promoted the 5mC modification of #13 in miR-200c-5p in colorectal cancer cells. Because the 5mC methylation rate is generally influenced by the activities of methylation/demethylation enzymes, our results imply the activities of 5mC methylation/demethylation enzymes are promoted/deactivated by m6A modifications in miR-200c-5p. As mentioned previously, cytosine is methylated in miR-200c-5p if it occurs in a motif recognized by NSUN2, suggesting that this m6A-dependent cytosine methylation may be caused by NSUN2. Therefore, we hypothesized that the NSUN2 protein has an amino acid sequence that recognizes m6A. We investigated amino acid homology between NSUN2 and YTHDF1, YTHDF2, and YTHDF3, which are known as m6A recognition proteins (Fig. 4d). We found that amino acids 535–578 of the NSUN2 protein have about 85% similarity with the YTH domain of the YTHDF protein family (Fig. 4e). Furthermore, NSUN2 also retains the amino acid (KS–WC) sequence^32^ that the YTHDF protein family requires for the recognition of m6A. These results suggest that NSUN2 has a YTH-like domain, which may promote cytosine methylation by recognizing m6A (Fig. 4f,g). To confirm the presence of NSUN2 in the vicinity of m6A, we performed the RNA immunoprecipitation using the NSUN2 antibody. The A-methylation rate (m6A/A) in 5mC-containing miRNA which binds NSUN2 was 27.4% (356/1302) (Fig. 4h: first right panel). On the other hand, A-methylation rate (m6A/A) in miRNA which neither contain 5mC nor bind NSUN2 was 12.7% (60/471) (Fig. 4h: second from the right panel). As expected, NSUN2-bound miRNAs tended to be high in m6A. Therefore, it was suggested that NSUN2 may recognize m6A and bind to RNA. Moreover, these results tend to be similar to those when measuring whole RNA (Fig. 4h: left two panels).

Overall, we measured the conductance profiles using a nano-fluid integrated nano-gap electrode device and successfully detected both A and C methylation sites simultaneously in sample RNA nucleotides extracted from cancer cell lines. The methylation positions were comparable to those determined by MALDI–TOF MS/MS. Furthermore, we evaluated the methylation ratios for each C and A site in the sequences and their relationship at the single-molecule level. These results suggest that the methylation ratio 5mC/C is facilitated by the presence of vicinal m6A methylation. This method is applicable for the comprehensive analysis of methylation site detection in the epitranscriptome, which will be useful for understanding these methylation events and their mechanisms, ushering in a new era in RNA biology.

## Methods

### Materials

Reagents and solvents were purchased from standard suppliers and used without further purification. Synthesized miRNAs were purchased from GeneDesign, Inc. Purification of oligonucleotides was performed on the Prominence High Performance Liquid Chromatograph (Shimadzu) using a COSMOSIL 5C_18_-MS-II packed column (4.6 mm I.D. × 150 mm, average particle size, 5 µm; Nacalai Tesque Inc.). Oligonucleotide concentrations were determined by UV absorbance at 260 nm using the NanoDrop ND-1000 UV–Vis Spectrophotometer (ThermoFisher Scientific). No buffer was added to the solutions of single nucleotides. A 1-mM phosphate buffer was used for preparation of all aqueous sample oligomer solutions.

### Cell culture and RNA extraction

DLD-1 cells were purchased from the American Type Culture Collection (Manassas). DLD-1 cells were cultured in Dulbecco’s Modified Eagle Medium (Cat. No. 08456-65; Nakarai Tesque Inc.) supplemented with 10% fetal bovine serum (Cat. No. 26140; ThermoFisher Scientific) at 37 ℃ in a humidified 5% CO_2_ atmosphere. Total RNA was extracted from cultured cells using the ISOGEN reagent (Cat. No. 311-02501; Nippongene) according to the manufacturer’s instructions.

### Fabrication of a device for miRNA detection

The nano-gap electrodes were constructed from nanofabricated mechanically controllable break-junctions (MCBJs) and the procedures for MCBJ fabrication are detailed elsewhere^33,34^. In this study, we utilized a nanochannel integrated nano-gap device and the detail device procedure are shown in the previous reports^22,24^. A polydimethylsiloxane (PDMS) cover was fused to the device substrate. The PDMS cover had a channel that connected the four hole for introducing the sample solution and the channel of the gap sensor. PDMS was purchased from Dow Corning Toray Co., Ltd. The electrophoresis electrodes were prepared by electrochemical oxidation of silver wires. A silver wire (The Nilaco Corporation) was electrochemically oxidized in 1 M NaCl using an electrochemical analyzer (Model 1030; ALS Co., Ltd.), and the resistance of the prepared Ag/AgCl electrode was around 20 kΩ. The formed gold nano-junction was broken by the home-made MCBJ systems^22,24^, and the sensing distance was set to 0.6–0.7 nm by the piezo element. During the measurement, the gap distance was maintained by feedback control of the piezo actuators.

### Test procedure

We formed a 0.8-nm electrode gap in a 0.10-μM target nucleotide solution in Milli-Q-purified water (Milli-Q model name/number; MilliporeSigma, Burlington). The current across the electrodes was recorded at 10 kHz using a custom-built logarithmic current amplifier and a PXI-4071 Digital Multimeter (National Instruments Corp.) under a DC voltage bias of 0.4 V. The applied voltage of the electrophoresis is +/-0.6V for each of the electrophoresis chambers in the device. After every 1 h of *I*–*t* measurement, we replaced the MCBJ sample with a new one. The measurements were carried out more than three times using different gold gap sensors.

### M6A containing miRNA immunoprecipitation

Small RNAs (< 100 nt) including miRNA were isolated from total RNA using the High Pure miRNA Isolation Kit (Cat. No. 05080576001; Sigma-Aldrich) according to the manufacturer’s instructions. Immunoprecipitation of m6A-containing small RNA was performed using an anti-m6A antibody (200 µg/ml) (Cat. No. 202003; Synaptic Systems) at 4 ℃ for 2 h. The m6A-containing small RNA–anti-m6A-antibody complexes were mixed with Dynabeads Protein G (Cat. No. 10003D, ThermoFisher Scientific) at 4 °C for 2 h. The mixture was isolated by magnetic separation. The m6A-containing small RNA was eluted from the mixture using 6.7 mM N6-methyladenosine 5-monophosphate sodium salt (Cat. No. M2780, Merck) at 4 °C for 2 h. The RNA not recovered by magnetic separation was designated as small RNA not containing m6A modifications.

### NSUN2 binding miRNA immunoprecipitation

Immunoprecipitation of NSUN2 binding small RNA was performed using an anti-NSUN2 antibody (Cat. No.20854-1-AP; Proteintech) at 4 ℃ for 2 h. The m6A-containing small RNA–anti-m6A-antibody complexes were mixed with Protein A/G PLUS-Agarose (Cat. No. sc-2033, Santa Cruz Biotechnology, inc.) at 4 °C for 2 h. The mixture was isolated by centrifugation at 12,000 rpm for 20 s. The NSUN2 binding RNA was reverse cross-linked by incubating tubes in a 67 °C water bath, mixing occasionally over two hours. Remove beads by centrifugation and continue incubating supernatant at 67 °C overnight. The RNA not recovered by centrifugation was designated as small RNA not binding to NSUN2.

### Amino acid homology analysis

Homology between the amino acid sequences of NSUN2, a cytosine methyltransferase, and YTHDF1, YTHDF2, and YTHDF3, which recognize m6A, was determined. The amino acid sequences of the proteins were obtained from NCBI Protein (NSUN2: NP_001180384.1; YTHDF1: NP_060268.2; YTHDF2: NP_057342.2; and YTHDF3: NP_001264746.1). For NSUN2, we used the sequence including amino acids 397–732; the RsmB domain was excluded from the analysis. The YTH domains of YTHDF1, YTHDF2, and YTHDF3 (YTHDF1: 390–559 aa; YTHDF2: 441–544 aa; YTHDF1: 366–499 aa) were selected from the entire amino acid sequence for analysis. The homology analysis was performed using GENETYX-MAC Ver. 18 (GENETYX CORPORATION).

### Matrix-assisted laser desorption/ionization–time-of-flight mass spectrometry (MALDI–TOF MS/MS)

Total RNA was hybridized with single-strand DNA oligonucleotides complementary to miR-200c-5p (5′-CCA AAC ACT GCT GGG TAA GAC G-3′) that were adenosine-methylated at the 5′ end via a C6 linker purchased from Hokkaido System Science Co., Ltd. The mixture was heated to 95 °C, then gradually cooled to 30 °C to anneal miR-200c-5p and the complementary DNA. The miR-200c-5p–DNA complex was incubated with Dynabeads M-270 Amine (Cat. No. 14307D, ThermoFisher Scientific) at 4 °C for 1 h. The mixture was heat-eluted and the supernatant obtained by magnetic separation. Lyophilized samples were used for subsequent experiments. Captured miR-200c-5p was purified using a ZipTip C_18_ cartridge column (Cat. No. ZTC18M96; MilliporeSigma) according to the manufacturer’s protocol. Purified miR-200c-5p was mixed with an aqueous solution of 3-hydroxypicolinic acid (Cat. No. 8201224; Bruker Daltonics) in a 1:1 (v/v) ratio, and 1 µl of the mixture was applied to an MTP AnchorChip 384 target plate (Cat. No. 8209514; Bruker Daltonics) and air-dried at room temperature. MALDI–TOF MS/MS analysis was performed using an ultrafleXtreme MALDI–TOF mass spectrometer (Bruker Daltonics) operated in negative-ion and reflectron modes. Spectra were manually acquired by FlexControl software v. 3.3.108.0 (Bruker Daltonics).

## Supplementary Information

Supplementary Information.

## References

[1] Li XY, Xiong XS, Yi CQ (2017). Epitranscriptome sequencing technologies: Decoding RNA modifications. Nat. Meth..

[2] Czerwoniec A (2009). MODOMICS: A database of RNA modification pathways: 2008 update. Nucleic Acids Res..

[3] Lo Monaco P, Marcel V, Diaz J-J, Catez F (2018). 2′-O-methylation of ribosomal RNA: Towards an epitranscriptomic control of translation?. Biomolecules.

[4] Krutyholowa R, Zakrzewski K, Glatt S (2019). Charging the code—tRNA modification complexes. Curr. Opin. Struct. Biol..

[5] Davalos V, Blanco S, Esteller M (2018). SnapShot: Messenger RNA modifications. Cell.

[6] Konno M (2019). Distinct methylation levels of mature microRNAs in gastrointestinal cancers. Nat. Commun..

[7] Yang Y, Hsu PJ, Chen Y-S, Yang Y-G (2018). Dynamic transcriptomic m^6^A decoration: Writers, erasers, readers and functions in RNA metabolism. Cell Res..

[8] Shen Q (2018). Tet2 promotes pathogen infection-induced myelopoiesis through mRNA oxidation. Nature.

[9] Eisenberg E, Levanon EY (2018). A-to-I RNA editing—immune protector and transcriptome diversifier. Nat. Rev. Genet..

[10] Eyler DE (2019). Pseudouridinylation of mRNA coding sequences alters translation. Proc. Natl. Acad. Sci. USA.

[11] Mauer J (2017). Reversible methylation of m^6^A_m_ in the 5' cap controls mRNA stability. Nature.

[12] Meyer KD (2012). Comprehensive analysis of mRNA methylation reveals enrichment in 3′ UTRs and near stop codons. Cell.

[13] Khoddami V (2019). Transcriptome-wide profiling of multiple RNA modifications simultaneously at single-base resolution. PNAS.

[14] Zhao BS, Roundtree IA, He C (2017). Post-transcriptional gene regulation by mRNA modifications. Nat. Rev. Mol. Cell Biol..

[15] Meyer KD, Jaffrey SR (2014). The dynamic epitranscriptome: N^*6*^-methyladenosine and gene expression control. Nat. Rev. Mol. Cell Biol..

[16] Rand AC (2017). Mapping DNA methylation with high-throughput nanopore sequencing. Nat. Methods.

[17] Branton D (2008). The potential and challenges of nanopore sequencing. Nat. Biotechnol..

[18] Lorenz DA, Sathe S, Einstein JM, Yeo GW (2020). Direct RNA sequencing enables m^6^A detection in endogenous transcript isoforms at base-specific resolution. RNA.

[19] Liu H (2019). Accurate detection of m^6^A RNA modifications in native RNA sequences. Nat. Commun..

[20] Gilbert WV, Bell TA, Schaening C (2016). Messenger RNA modifications: Form, distribution, and function. Science.

[21] Ohshiro T (2012). Single-molecule electrical random resequencing of DNA and RNA. Sci. Rep..

[22] Di Ventra M, Taniguchi M (2016). Decoding DNA, RNA and peptides with quantum tunnelling. Nat. Nanotechnol..

[23] Ohshiro T, Tsutsui M, Yokota K, Taniguchi M (2018). Quantitative analysis of DNA with single-molecule sequencing. Sci. Rep..

[24] Ohshiro T (2019). Direct analysis of incorporation of an anticancer drug into DNA at single-molecule resolution. Sci. Rep..

[25] Zwolak M, Di Ventra M (2005). Electronic signature of DNA nucleotides via transverse transport. Nano Lett..

[26] Lagerqvist J, Zwolak M, Di Ventra M (2006). Fast DNA sequencing via transverse electronic transport. Nano Lett..

[27] Ewing B, Green P (1998). Base-calling of automated sequencer traces using *phred*. II. Error probabilities. Genome Res..

[28] Ewing B, Hillier L, Wendl MC, Green P (1998). Base-calling of automated sequencer traces using *phred*. I. Accuracy assessment. Genome Res..

[29] Hur K (2013). MicroRNA-200c modulates epithelial-to-mesenchymal transition (EMT) in human colorectal cancer metastasis. Gut.

[30] Yang X (2017). 5-methylcytosine promotes mRNA export—NSUN2 as the methyltransferase and ALYREF as an m^5^C reader. Cell Res..

[31] Liu JZ (2014). A METTL3-METTL14 complex mediates mammalian nuclear RNA *N*^6^-adenosine methylation. Nat. Chem. Biol..

[32] Xu C (2015). Structural basis for the discriminative recognition of *N*^6^-methyladenosine RNA by the human YT521-B homology domain family of proteins. J. Biol. Chem..

[33] Tsutsui M, Shoji K, Taniguchi M, Kawai T (2008). Formation and self-breaking mechanism of stable atom-sized junctions. Nano Lett..

[34] Agrait N, Yeyati AL, van Ruitenbeek JM (2003). Quantum properties of atomic-sized conductors. Phys. Rep..

