# Supplementary material for: Single-molecule RNA sequencing for simultaneous detection of m6A and 5mC
Source: Sci Rep. 2021 Sep 29;11:19304. doi: 10.1038/s41598-021-98805-z (PMC8481274; doi:10.1038/s41598-021-98805-z)
Supplement: Supplementary file 1 — Supplementary Information. [file 41598_2021_98805_MOESM1_ESM.pdf]

Single-Molecule RNA Sequencing for Simultaneous Detection of m6A and 5mC

Takahito Ohshiro[1†], Masamitsu Konno[2†], Ayumu Asai[2,3†], Yuki Komoto[2], Akira Yamagata[2,4], Yuichiro Doki[5], Hidetoshi Eguchi[5], Ken Ofusa[2,4], Masateru Taniguchi[1], Hideshi Ishii[2*]*

Affiliations

1. *The Institute of Science and Industrial Research, Osaka University, 8-1 Mihogaoka, Ibaraki, Osaka, 567-0047, Japan.*
2. *Center of Medical Innovation and Translational Research, Graduate School of Medicine, Osaka University, 2-2 Yamadaoka, Suita, Osaka, 565-0871, Japan*
3. *Artificial Intelligence Research Center, The Institute of Scientific and Industrial Research, Osaka University, 8-1 Mihogaoka, Ibaraki, Osaka, 567-0047, Japan.*
4. *Prophoenix Division, Food and Life-Science Laboratory, Idea Consultants, Inc., 1-24-22 Nanko-kita, Suminoe-ku, Osaka-city, Osaka, 559-8519, Japan*
5. *Gastroenterological Surgery, Department of Surgery, Graduate School of Medicine, Osaka University, 2-2 Yamadaoka, Suita, Osaka, 565-0871, Japan*

Table of Contents

S1. Signal measurement for mono-nucleotide and its conductance profiles·········· 2
S2. Signal picking and analysis·· 3
S3. Mononucleotide signal detection·· 4
S4. Background and Noise level for methylated/non-methylated nucleotide signals in the single molecule conductance profiles ··· 6
S5. Sensitivity and specificity for methylated/non-methylated nucleotide in single-molecule detection ·· 8
S6. Base calling··· 11
S7. Signal assembly and conductance plotting/mapping····································· 12
S8. Synthetic methylated RNA oligonucleotides ··· 13
S9. Determination ratio of Mixed methylated/non-methylated RNA nucleotides·····17
S10. Methylation detection of MALDI–TOF mass spectrum ···································18

S1. Signal measurement for mono-nucleotide and its conductance profiles

For the RNA used here, we measured the natural RNA nucleotide of Adenosine monophosphate (rAMP), cytidine monophosphate (rCMP), Guanosine monophosphate (rGMP), and uridine monophosphate (rUMP), and the single molecules of 5mC and m6A used in this study. As a result, we obtained the following conductance-time profile signals (Figure S1).

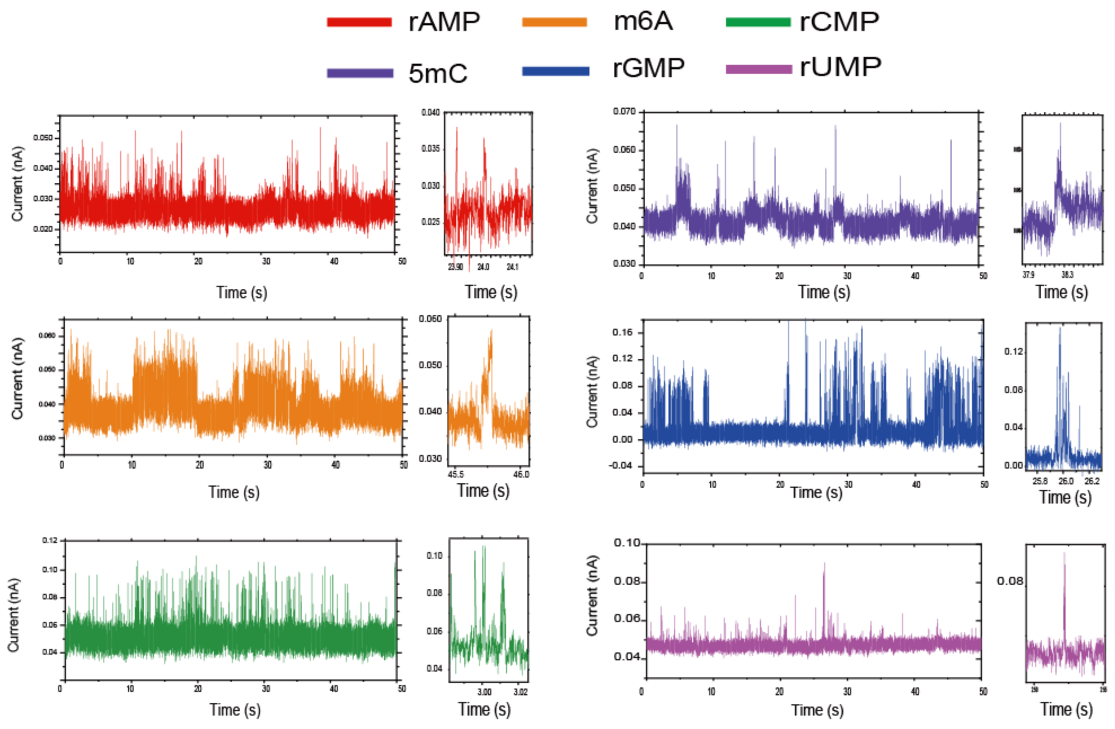

Figure S1. Mononucleotide signal detection. Single-molecule electrical signals for rAMP (red), rCMP (green), rGMP (blue), rUMP (pink), m6A (orange) and 5mC (purple)are measured by using a 0.64-nm gap electrode, which was tuned by a nano-fabricated mechanically controllable break junction (nano-MCBJ).

S2. Signal picking and analysis

From the obtained conductance-time profile, we picked single-molecular signals up as follows: First, each of the signal “start-time” was determined from the conductance-time profiles. The signal “start-time” is defined as the conductance data-point when the current data exceeds the certain current level (“rise-threshold”) from the estimated “baseline”. In this study, the decision of “rise-threshold” was set to be six times the “noise” level. The “baseline” is defined as the mode of the histogram for the last 2000 data points at each time point. The “noise” is defined as the mode of the standard deviation of 200 data points obtained by dividing the latest 10,000 data points at each time point into 500 equal parts. Second, the signal “end-time” is determined from the conductance-time profiles after the signal “start-time”. The signal “end-time” is defined as the data point below the certain current level (“fall-threshold”) from the estimated baseline. In this study, the “fall-threshold” is set to be one times the “noise” level. Based on this signal picking algorithm, all the signal regions for mono-nucleotide and oligonucleotide are extracted from conductance-time profiles, and these obtained signals were used for the following signal identification and conductance plotting procedures.

S3. Mononucleotide signal detection

Single-molecule electrical signals for m6A and 5mC are measured by using a 0.64-nm gap electrode, which was tuned by a nano-fabricated mechanically controllable break junction (nano-MCBJ). **Figure S2a** shows typical *I-t* profiles for 5mC in aqueous solution. The observed electrical signals were characterized by I_p, which were defined as the maximum current and the duration of the current, respectively. Single-molecule conductance (I_p/V) histograms were then constructed from the I_p data using approximately 1000 signals for each molecule and were analyzed using Gaussian fit curves. We defined the peak values of the Gaussian fit curves as the single-nucleotide conductance. The conductance of 5mC was found to be 149 pS compared with 64 pS for cytidine **(Figure S2b**). Similarly, the single-nucleotide conductances were found to be 111 pS for m6A compared with 92 pS for adenosine (**Figure S2c**). The conductance values determined by this single-molecule electrical detection are closely related to characteristic energy levels, particularly the HOMO energy level, which is calculated by density functional theory. The conductance differences caused by methylation for the pyrimidine, i.e., C and 5mC, and purine, i.e., A and m6A, are due differences in the π-conjugation induced by the electron-donating character of methyl substitutions. Together with these data, the order of conductance values order was found to be the following: 5mC (149 pS)>G (123 pS)>m6A (111 pS)>A (92 pS)>C (64 pS)>rUMP (50 pS). The values relative to G, which are the values normalized to the G conductance value (123 pS) were ordered as follows: 5mC (1.21)>rGMP (= 1)>m6A (0.90)>rAMP (0.77)>rCMP (0.52)>rUMP (0.41) (Table 1).

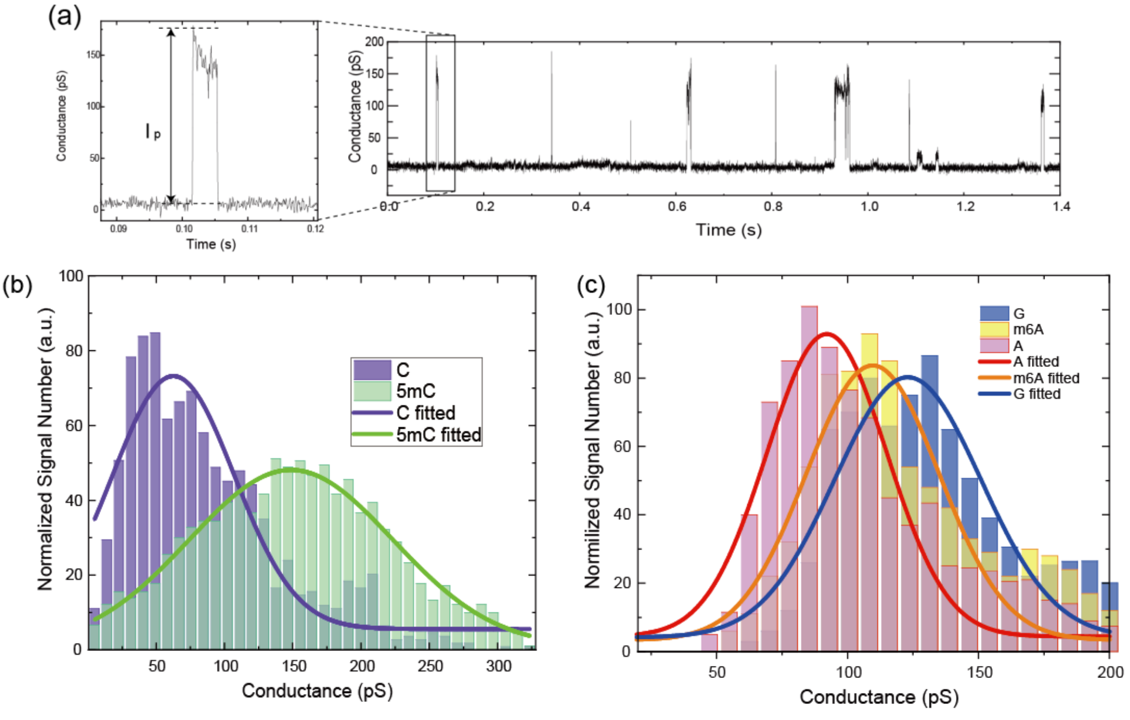

Figure S2. Mononucleotide signal detection. (a) Typical *I*-*t* profiles for 5mC in aqueous solution. The observed electrical signals were characterized by I_p, which were defined as the maximum current and the duration of the current, respectively. (b) The conductance histogram for C and 5mC. The conductance of 5mC was found to be 149 pS compared with 64 pS for cytidine. (c) The conductance histogram for m6A and A and G. The conductance of m6A was found to be 111 pS compared with 123 pS for A and 92 pS for A. The conductance values determined by this single-molecule electrical detection are closely related to characteristic energy levels, particularly the HOMO energy level, which is calculated by density functional theory. Together with these data, the order of conductance values order was found to be the following: 5mC (149 pS)>G (123 pS)>m6A (111 pS)>A (92 pS)>C (64 pS)>rUMP (50 pS). The values relative to G, which are the values normalized to the G conductance value (123 pS) were ordered as follows: 5mC (1.21)>rGMP (= 1)>m6A (0.90)>rAMP (0.77)>rCMP (0.52)>rUMP (0.41) (Table 1).

S4. Background and Noise level for methylated/non-methylated nucleotide signals in the single molecule conductance profiles

The noise and baseline for DNA nucleotide and oligonucleotide in the single molecule sequencer so far are shown in the previous studies. Similarly, we demonstrated the conductance-time profiles for rAMP, rGMP, rUMP, rGMP, 5mC, and m6A solutions in the no-signal region. The "baseline" is defined as the mode of the histogram for the last 2000 data points at each time point. The "baseline" level was found to be in the range from 2 pA to 100 pA. The "noise" is defined as the mode of the standard deviation of 200 data points obtained by dividing the latest 10,000 data points at each time point into 500 equal parts. The "noise" level was found to be in the range from 2 pA to 10 pA. There is no significant difference in noise and baseline levels among the RNA nucleotide types used here. In order to evaluate the signal sensitivity, it is generally used the ratio of the signal intensity to the noise level (S / N ratio). In this study, in order to secure the signal sensitivity, the signals with an S / N ratio of over six were used for the following signal analysis.

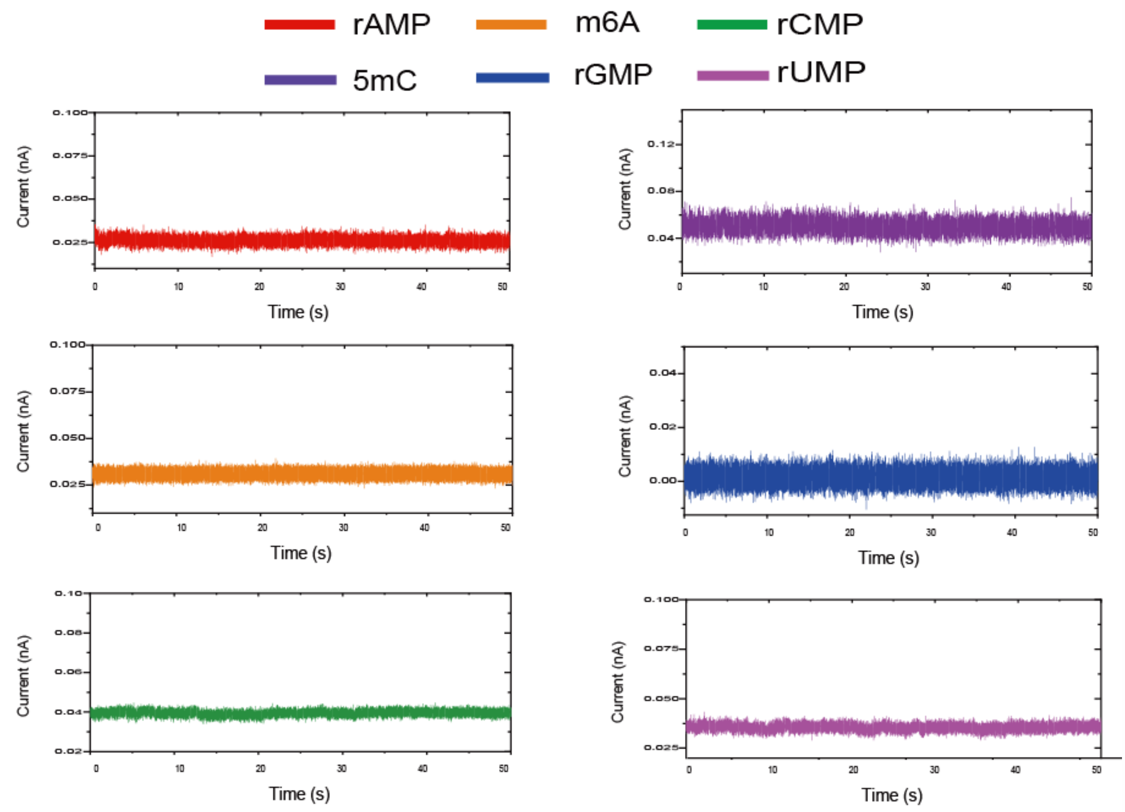

Figure S3. Noise signal and background current. Typical noise and background current profiles for sample solution of rAMP (red), rCMP (green), rGMP (blue), rUMP (pink), m6A (orange) and 5mC (purple) are measured by using a 0.64-nm gap electrode, which was tuned by a nano-fabricated mechanically controllable break junction (nano-MCBJ).

S5. Sensitivity and specificity for methylated/non-methylated nucleotide in single-molecule detection

Each of the number of signals acquired by the above mentioned signal measurement and its signal extraction method was found to be 132,125 for rCMP, 81,456 for rAMP, 45,829 for rGMP, 88,339 for rUMP, 41,213 for 5mC, and 12,441 for m6A, respectively. In order to identify the nucleotide-species, based on the signals of the measured conductance profiles, we statistically compared methylated nucleotides (m6A, and 5mC) with unmodified nucleotides (A, and C). From signal conductance-time profiles, several kinds of the feature signal values, the maximum value of the conductance, the duration of the signal, and the standard deviation of the conductance, and signal shape factors, were obtained. The detailed estimation procedure of the signal-shape factors is shown in the next section. These signal feature values reflected the difference between the methylated and non-methylated nucleotides. For instance, the histogram of the maximum value of the conductance profile demonstrated the characteristic conductance values for each of the nucleotides (**Figure S2 b and c**). From the previous study, it was found that the difference in the maximum value of the conductance profile is closely related to the HOMO level of the nucleotide molecules.

In order to evaluate the sensitivity and specificity from the single-molecule conductance profiles, we used the F-measure for the distinction accuracy between non-methylated and methylated nucleotides. In statistical analysis of binary classification (for instance, methylated and non-methylated nucleotides), the F-measure is a measure of a test's accuracy. It is calculated from the precision and recall of the test, where the precision is the number of true positive results divided by the number of all positive results, including those not identified correctly, and the recall is the number of true positive results divided by the number of all samples that should have been identified as positive. Precision is also known as positive predictive value, and recall is also known as sensitivity in diagnostic binary classification.

The identification procedure is as follows. For the feature parameters used in this study, we used thirteen features parameters as follows; the peak value of the signal current (I_p), the average signal value (I_{ave}), the duration-time (t_d), and the twelve-dimensional shape factor (S_1, S_2, S_3, S_4, S_5, S_6, S_7, S_8, S_9, S_{10}, S_{11}, S_{12}). The twelve-dimensional shape factor values are defined as shown as follows. Briefly, a current-time (I-t) profile of a signal region is separated into ten time-regions, and then each of the average current value (blue circle) for each of the time regions (I_1, I_2, I_3, I_4, I_5, I_6, I_7, I_8, I_9, I_{10}) is calculated. Each of the current values was normalized by the maximum current value of the signal (I_p), and the values in each region were defined as the shape factor (S_1, S_2, S_3, S_4, S_5, S_6, S_7, S_8, S_9, S_{10}, S_{11}, S_{12}). In the third step, each of the extracted signals is converted into these features

data (I_p, I_{ave}, t_d, S_1, S_2, S_3, S_4, S_5, S_6, S_7, S_8, S_9, S_{10}, S_{11}, S_{12}). The dataset of the features data is divided into two datasets, i.e., “training” data and “test” data. In the fourth step, a machine learning classifier learns these feature parameters from the “training” data. In the fifth step, the classifier is utilized to identify each of the nucleotide species of the signals for the “test” data, and the discrimination accuracy is evaluated as F-measure values. In the final step, a validation of the accuracy was evaluated by performing ten-fold cross-validation.

In this two-class problem (methylated or no-methylated), we can assign the signals obtained in the methylated nucleotide solutions as “true methylated signals” and the signals obtained in the non-methylated nucleotide solutions as “true non-methylated signals. On the other hand, by using the classifier as mentioned above, we can predict the ‘predicted’ methylated signals or ‘predicted’ methylated signals for test signals. Therefore, all the test signals were categorized to four groups; ‘correctly predicted and true methylated (true positive)’, ‘correctly predicted and true no-methylated (true negative)’, ‘predicted methylated but true no-methylated (false negative)’, ‘predicted non-methylated but true methylated (false positive)’, where “true signals” means correctly predicted methylated nucleotide species, “false signals” means incorrectly predicted event values. These four values are summarized as a confusion matrix. In this study, the accuracy of methylation/non-methylation judgments by the cross-validation of the test data are shown in a confusion matrix of Figure S4a for 5mC/C and S4b for m6A/A, respectively. From these classification metrics such as precision, recall, specificity and sensitivity of our classifier, the F-measure for methylated (m6A) and non-methylated (A) of adenosine was found to be 0.88. Similarly, the F-measure for methylated (5mC) and non-methylated (C) of cytidine is 0.85.

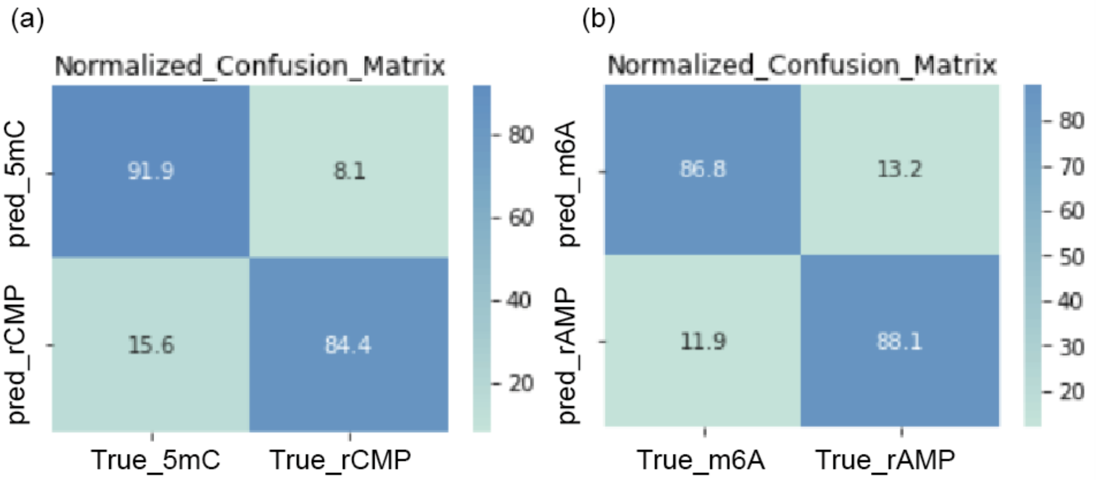

Figure S4. Accuracy of methylation and non-methylation for 5mC/C and m6A/A .

Based on these F-measure values, we calculated the determination accuracy for the discrimination between methylated cytosine (5mC) and unmodified cytosine (C), and between methylated adenosine (m6A) and non-methylated adenosine (A) of A. The accuracy of a determined base (Pr) is calculated on the following equation (Ref. Churchill, G. A. & Waterman, M. S. The accuracy of DNA sequencers: Estimating sequences quality. Genomics.14, 89-98, (1992).).

$$Pr = 1 - exp\left\{-\left(\frac{p}{1-p}\right)^{d}\right\} \quad .....(1)$$

The accuracy of a determined base (Pr) is determined by the signal count number (d), which is defined as the sum of all the counts of methylated and non-methylated signals, and the assigned accuracy (p) of each base. According to this equation, increasing the count, d, increases the accuracy of the determined base species. Each of the number of signals acquired by the above mentioned signal measurement and its signal extraction method was found to be 132,125 for rCMP, 81,456 for rAMP, 45,829 for rGMP, 88,339 for rUMP, 41,213. When using the F-measure of 0.88 for adenosine methylation (m6A/A) and the F-measure of 0.85 for cytidine methylation (5mC/C) as the assigned accuracy (p), the error probability was found to be almost one. Therefore, this quantitative evaluation is robust enough in this study.

S6. Base calling

The base sequences from the conductance profiles were determined by a Phred base-calling method, which is widely used for conventional genome sequencer analysis[S1,S2]. In the case of the conventional sequencer, base calling is based on the optical intensity for each wavelength of the base-attached optical probes. In our case, base calling was performed based on the tunnel-current intensity; the detailed procedure is described in previous reports[23] and is only briefly described here.

In the first step, the data histograms were derived from the conductance–time profiles, and each peak value of the conductance (μ) and standard deviation (σ) for each base is determined by the Gaussian fitting of the datapoint histogram. In each conductance-profile histogram, there are several peaks. Among them, the minimum and maximum values of the peaks correspond to the relative conductance values of baseline and G, respectively (Table 1). In the second step, the peak conductance and the standard deviation values were used to calculate each base probability and each base element. In the third step, base species were sequentially assigned in the time profiles based on the maximum probability values of each base type or the baseline. For this assignment, the integral value of the base probability for each length of time was calculated to reduce the abrupt signal change, which was mainly due to electrical noise. In this study, we set the time length for integration to 0.3 msec, which is comparable to the experimentally determined minimum retention time around the electrodes. The maximum integral value of each base-probability value was sequentially assigned to the base type or baseline for each of the time regions. For each of the assigned bases, the accuracy of the base assignment was quantified by the Q score[S2]. Signals with low Q values were due to the large conductance distributions and their overlap. To ensure the accuracy of read-sequence signals, only read-sequence signals with over six Q scores (P>75%) were used for subsequent resequencing.

S7. Signal assembly and conductance plotting/mapping

Signal assembly was performed based on tunnel-current intensity, and the detailed procedure has been described previously[22]; the procedure is only briefly described here. A sequence position number was assigned for each read base in the "long-read signals" and compared to the miRNA-200c-5p reference sequence; over six right-read base signals were described as "long-right read signals" and used for assembly and plotting. The read direction was sometimes found to be changed in the terminal position, and therefore a "duplicated" read was observed. In the assembly process, these duplicated-read regions were automatically deleted after sequence-position assignment, and the straight-right read regions were used. For the assembly, we used the miRNA-200c-5p reference sequence and all the methylated derivatives from all the signals, and the resulting conductance profiles of all the long-read signals are shown in **Figs. 2 a–c**.

The signal count of methylated and non-methylated bases for each base position were performed as follows. In the case of position #4 in miRNA-200c-5p (**Fig. 3a**, first column), we used the miRNA-200c-5p reference sequence and all the methylated derivatives determined from the obtained signals; of these, signals containing cytidine (C) and methylated cytidine (5mC) in the #4 position of miRNA-200c-5p were determined from the captured miRNA sample solution. From the signals for the captured miRNA-200c-5p, we extracted all the over six right-read base signals containing cytidine (C) and methylated cytidine (5mC) in the #4 position of miRNA 200c-5p, and the total signal number was found to be 1606. The number of signals for 5mC and C were 10 and 1596, respectively, so that the methylation rate was 0.6% (10/1606). We used a similar method for determining the methylated signal number and its methylation rate for all the potential methylated positions (Figs. 3a and 3b and Figs. 4a and 4b).

S8. Synthetic methylated RNA oligonucleotides

In previous studies, synthetic nucleotide samples have been used to identify the sequence of miRNA, e.g., hsa-let7a-5p, hsa-let7c-5p, hsa-let7e-5p, hsa-let7f-5p and, so on., and determine the sample nucleotide ratio in mixed miRNA solutions [22]. Similarly, in this study, we performed similar identification for synthetic non-methylated nucleotides, 5'-CGCUGCU-3', and synthetic methylated nucleotide, 5'-CGmCUG mCU-3', in which cytosine at positions #3 and #6 was methylated as 5-methylated cytosine in the 5'-CGCUGCU-3'. We obtained conductance-time profiles (**Fig S5**), by using this single-molecule electrical detection method with nanochannel devices. The obtained conductance-time profiles represent the characteristic conductance for each single-molecule conductance value (**Table 1**). The relative conductance was found to be 1.0 for G, 0.58 for C, mC (5mC) for 1.21, and 0.41 for U, respectively. Actually, in the obtained conductance-time profiles, characteristic conductance levels were observed. For instance, in the figure SX1, the five conductance levels (orange, blue, purple, green, and black line) were observed. Based on the single-molecule conductance values, orange, blue, purple, green were corresponding to the 5mC, G, C, and U, respectively while the black line were corresponding to baseline current levels. (**Table 1**),

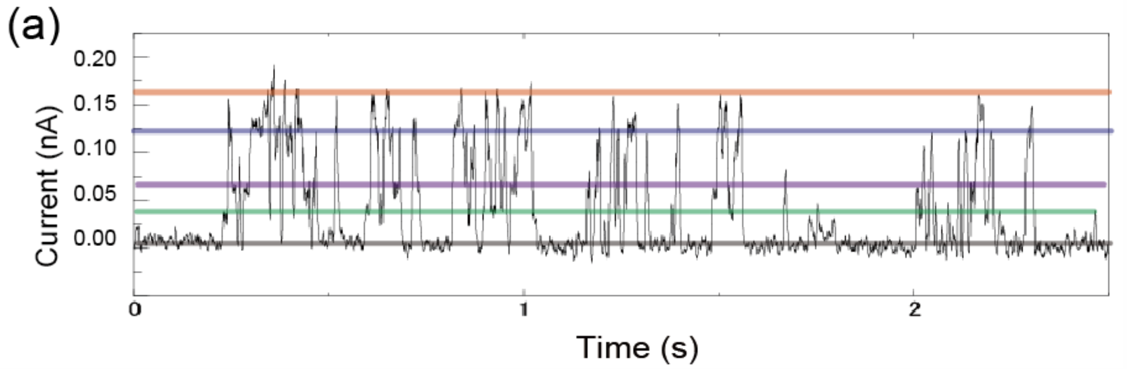

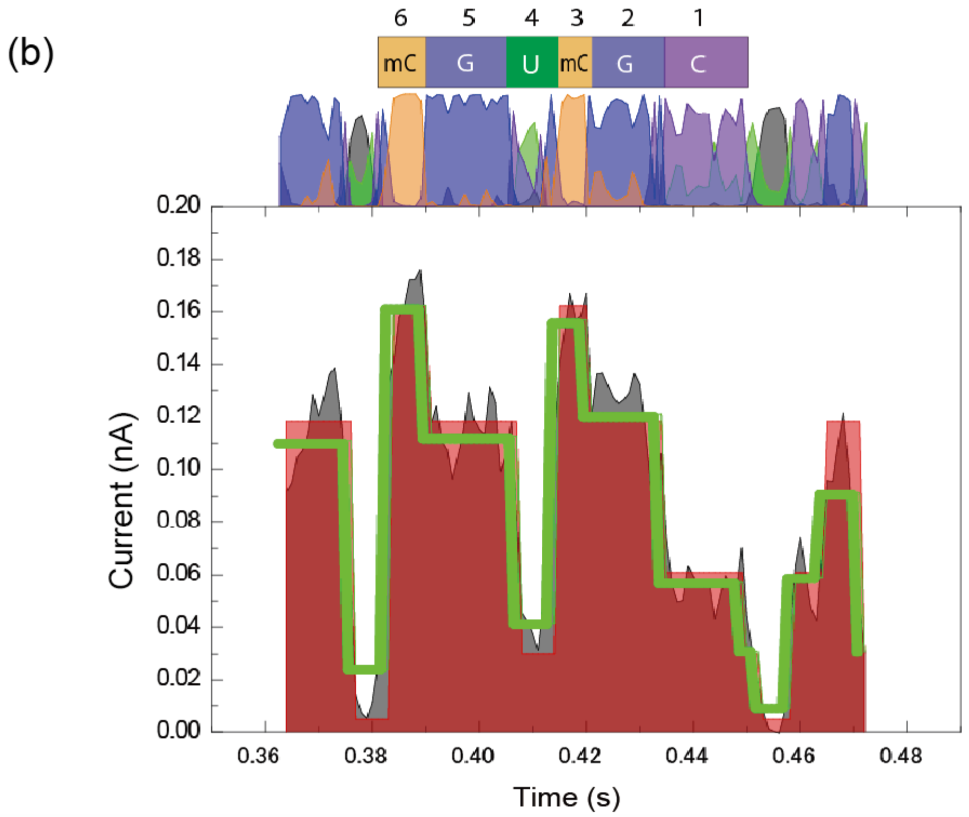

Figure S5. (a) Synthetic methylated RNA oligonucleotides (5'-CGmCUG mCU-3') . Typical noise and background current profiles. **(b) Base-calling of Fragmented RNA methylated RNA oligonucleotides (5'-CGmCUG mCU-3').** By using our base-calling method, we can determine the partial sequence of conductance profiles as 5-CGmCUmC. The relative conductance values (Table 1) were used to calculate each base probability and each base element. The base species were sequentially assigned in the time profiles based on the maximum probability values of each base type or the baseline.

For this conductance profile, the probability density at each time is calculated based on character parameters of the conductance profiles including the characteristic value of the single molecule conductance in Table 1. Based on the base type of the maximum probability value at each time, the base sequence of the conductance-profile was determined (**Fig S5b**).

Based on this fragment sequence, each of the conductance plots are plotted as shown in **Fig S6a**, which was found to be corresponding to the base position of the original sequence (5'-CGmCUGmCU-3'). Similarly, each of the conductance plots are plotted as shown in **Fig S6b**, which was found to be corresponding to the base position of the original sequence (5'-CGCUGCU-3') . In addition, in order to check detection ability for the RNA containing five kinds of nucleotide species (adenosine, guanosine, cytidine, uridine, methylated cytidine), we performed single-molecule electrical detection of non-

methylated RNA (5’-CUU UCC CGG AAU ACG CCC AGA UGA G-3’) and methylated RNA (5’-CUU UCC CGG AAU AmCG CCC AGA UGA G-3’), and obtained the conductance plots (**Fig S6c and d**). This suggests that it is possible to show that RNA methylation identification is possible even by single molecule measurement.

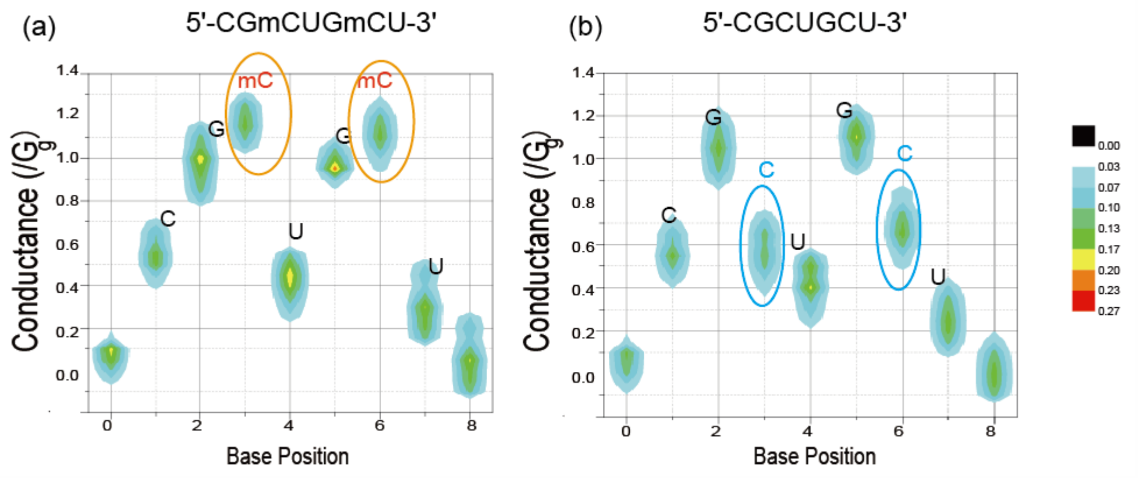

Figure S6 (a, b). Conductance plot for methylated RNA (5'-CGmCUGmCU-3') and non-methylated RNA (5'-CGCUGCU-3').

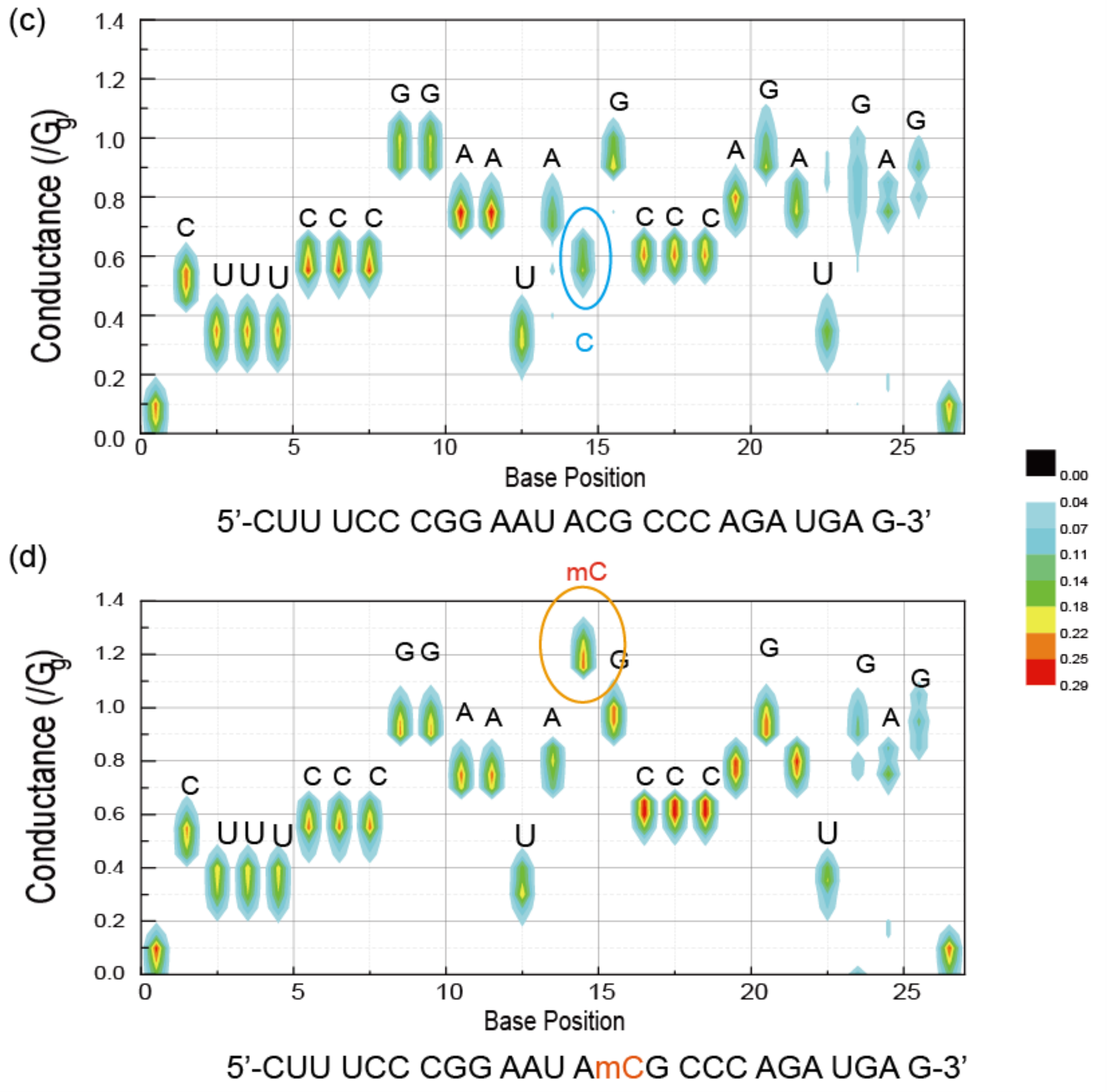

Figure S6 (c, d). Conductance plot for methylated RNA (5'-CUU UCC CGG AAU AmCG CCC AGA UGA G-3') and non-methylated RNA (5'-CUU UCC CGG AAU ACG CCC AGA UGA G-3')

S9. Determination ratio of Mixed methylated/non-methylated RNA nucleotides

This methylation ability was also utilized for the determination of methylation ratio in the mixed discrimination of the methylation discrimination of # 7 and # 13 of the synthetic miR-200c-5p. For identification, the identification learner shown above was carried. The mixing ratio of methylated adenosine (m6A) and non-methylated adenosine (A) was 5%, 20%, 40%, 60%, 80%, 95%, indicating the ratio of methylated molecules to the total number of molecules counted (**Figure S7a**). Similarly, the mixing ratio of methylated cytosine (5mC) and non-methylated cytosine (C) was 5%, 20%, 40%, 60%, 80%, 95%, indicating the ratio of methylated molecules to the total number of molecules counted (**Figure S7b**). This indicates that it is also possible to identify methylated mixture in the RNA sequence.

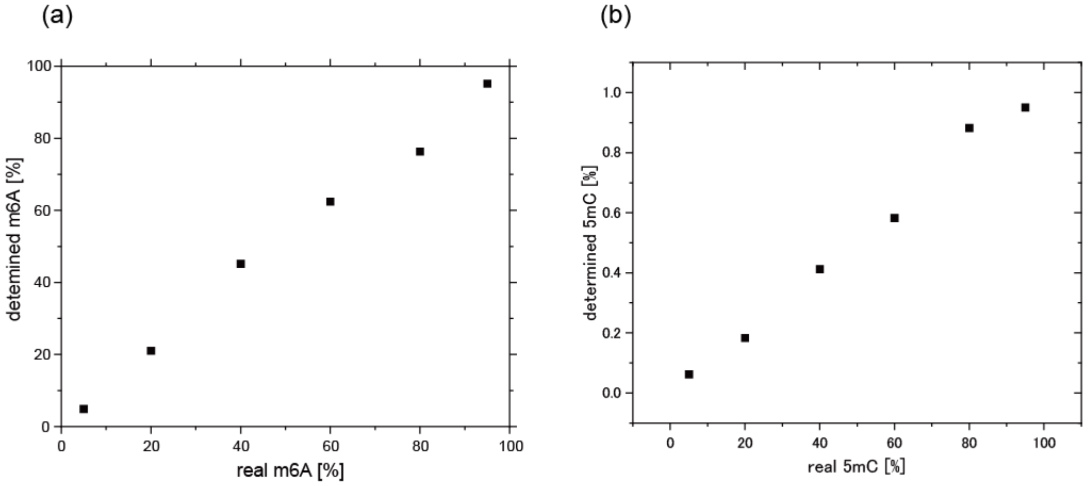

Figure S7 (a, b). Comparison of the original sample concentration ratio and the determined concentration ratio for m6A/A mixed signals (a) and 5mC/C mixed signals (b).

S10. Methylation detection of MALDI–TOF mass spectrum

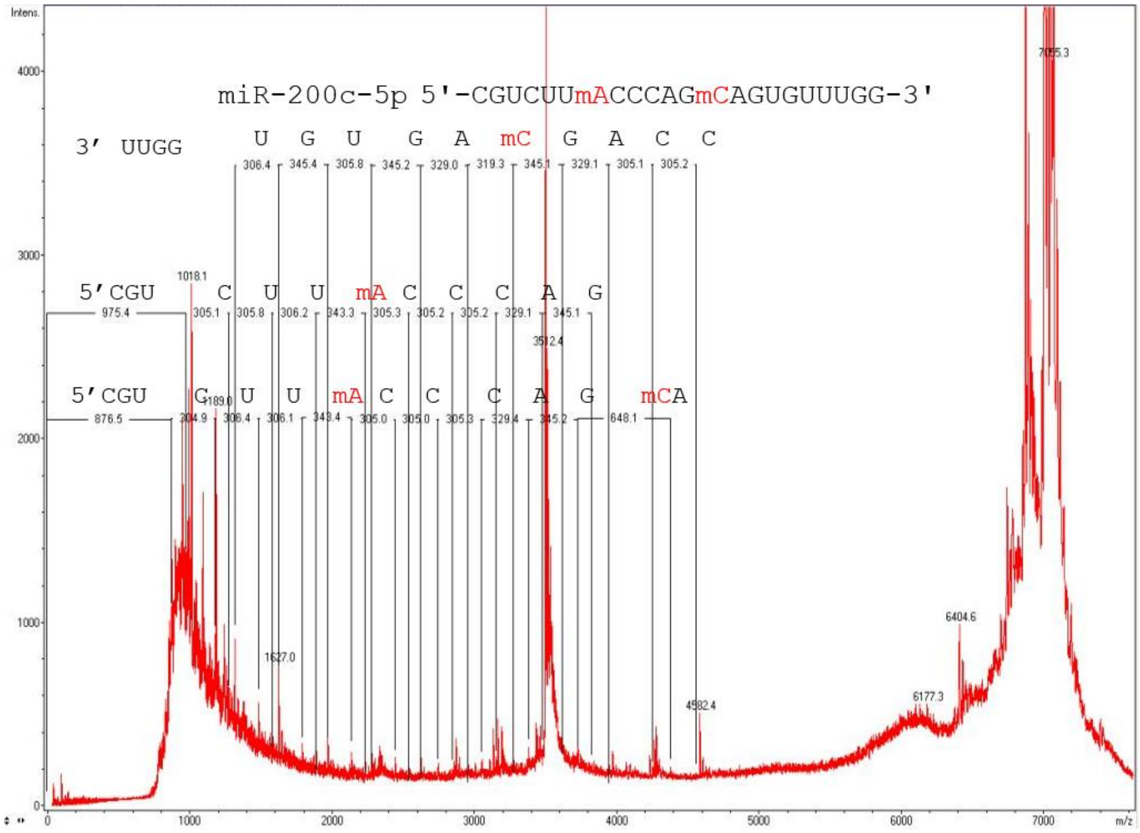

Figure S8. Methylation detection of MALDI–TOF–MS/MS spectrum of miR-200c-5p. Fragment ions from base 4-9, 4-10, and 18-9 are shown. The adenosine at position 7 and the cytidine at position 13 show an additional peak (+14 m/z), indicating methylation (m6A and 5mC).

Supporting information references

[S1]. Ewing, B. & Green, P. Base-calling of automated sequencer traces using phred. II. Error probabilities. *Genome Res.* **8**, 186–194 (1998).

[S2]. Ewing, B., Hillier, L., Wendl, M. C. & Green, P. Base-calling of automated sequencer traces using phred. I. Accuracy assessment. *Genome Res.* **8**, 175–185 (1998).
